# The Importance of Total Neutrophil Count and Neutrophil/Lymphocyte Ratio in ST Elevation Myocardial Infarction

**DOI:** 10.15171/jcvtr.2014.024

**Published:** 2014-12-30

**Authors:** Mehmet Agilli, Ibrahim Aydin, Fevzi Nuri Aydin, Tuncer Cayci, Yasemin Gulcan Kurt

**Affiliations:** ^1^Department of Biochemistry, Agri Military Hospital, Agri, Turkey; ^2^Department of Biochemistry, Sarikamis Military Hospital, Sarikamis, Kars, Turkey; ^3^Department of Biochemistry, Sirnak Military Hospital, Sirnak, Turkey; ^4^Department of Medical Biochemistry, Gulhane Military Medical Academy, Ankara, Turkey

## Dear Editor,

We have read with a great interest the published article by Ghaffari et al. entitled with “The predictive value of total neutrophil count and neutrophil/lymphocyte ratio in predicting in-hospital mortality and complications after STEMI”.^1^ Authors have suggested that complete blood count (CBC) test might help to identify ST elevation myocardial infarction (STEMI) patients and higher neutrophil count had the best predictive value for both mortality and heart failure. However, we think there are some points that should be mentioned as contributory factors.

Total neutrophil (TN) and neutrophil/lymphocyte ratio (NLR) are easily calculable laboratory parameters used to evaluate systemic inflammation. It was shown that diabetes mellitus, essential hypertension, metabolic syndrome, smoking, obesity, thyroid disorders, inflammatory bowel disease, renal and/or hepatic failure, and many inflammatory diseases may potentially affect TN and NLR.^2-4^ The authors have defined exclusion criteria as active inflammation or chronic inflammatory diseases, past history of surgery within 3 months prior to MI, and cancer. But above TN/NLR-affecting factors such as hypertension, diabetes mellitus, smoking and hyperlipidemia were included to the study. Therefore, in our opinion, assessment of TN and NLR could not provide reliable information.

NLR are easily obtained by CBC analysis which is being used almost all clinical laboratories. The authors have not defined which analyzer or analyzers were used for this aim. It should not be ignored the fact that the different analyzers could provide different results. In addition, it would have been better if the authors indicated the elapsed time between obtaining the blood samples and measuring NLR, because pre-analytical waiting period could affect these parameters.^5^

We believe that the findings of Ghaffari et al. will lead to further studies about the value of TN and NLR upon in-hospital death and complications after a documented STEMI. On the other hand, explanation of these concerns would certainly provide to the readers clearer information.

## Ethical issues

Not applicable.

## Competing interests

Authors declare no conflict of interests in this study.

## References

[1] Ghaffari S, Nadiri M, Pourafkari L, Sepehrvand N, Movasagpoor A, Rahmatvand N (2014). The predictive Value of Total Neutrophil Count and Neutrophil/Lymphocyte Ratio in Predicting In-hospital Mortality and Complications after STEMI. J Cardiovasc Thorac Res.

[2] Tanoglu A, Karagoz E, Yiyit N, Berber U (2014). Is combination of neutrophil to lymphocyte ratio and platelet lymphocyte ratio a useful predictor of postoperative survival in patients with esophageal squamous cell carcinoma?. Onco Targets Ther.

[3] Shiny A, Bibin YS, Shanthirani CS, Regin BS, Anjana RM, Balasubramanyam M (2014). Association of Neutrophil-Lymphocyte Ratio with Glucose Intolerance: An Indicator of Systemic Inflammation in Patients with Type 2 Diabetes. Diabetes Technol Ther.

[4] Campesi I, Carru C, Zinellu A, Occhioni S, Sanna M, Palermo M (2013). Regular cigarette smoking influences the transsulfuration pathway, endothelial function, and inflammation biomarkers in a sex-gender specific manner in healthy young humans. Am J Transl Res.

[5] Imeri F, Herklotz R, Risch L, Arbetsleitner C, Zerlauth M, Risch GM (2008). Stability of hematological analytes depends on the hematology analyser used: a stability study with Bayer Advia 120, Beckman Coulter LH 750 and Sysmex XE 2100. Clin Chim Acta.

